# Electropolymerization of Silver(I) Helicate into Conductive Metallopolymer: Structural and Functional Insights

**DOI:** 10.1021/acs.inorgchem.5c02774

**Published:** 2025-10-17

**Authors:** Sergiusz Napierała, Maciej Kubicki, Monika Wałęsa-Chorab

**Affiliations:** Faculty of Chemistry, 467899 Adam Mickiewicz University in Poznan, Uniwersytetu Poznanskiego 8, Poznan 61-614, Poland

## Abstract

A novel luminescent dinuclear helicate silver­(I) complex has been synthesized via a complexation reaction with a N_4_-donor ligand containing two triphenylamine (TPA) moieties. The structure of the complex has been confirmed by X-ray diffraction analysis and it revealed the presence of argentophilic interactions. The photophysical studies confirmed that ligand **L**, as well as the Ag­(I) complex, is luminescent in the solid state. The Ag­(I) complex exhibited a sharp emission band in the blue region from ligand molecules and broad emission band in the range 475 nm–700 nm assigned to ligand-to-metal charge transfer transition (LMCT) perturbed by the presence of argentophilic interactions. Both free ligand **L** and its Ag­(I) complex undergo electropolymerization, leading to the formation of thin films. Moreover, electropolymerization of the Ag­(I) complex occurred more easily compared with ligand **L**. The resulting polymer exhibited distinct spectroelectrochemical properties, enhanced stability, and 2-step color change from greenish yellow via orange to blue upon redox switching with fast coloration/bleaching times (2.7 s). These investigations demonstrate that the silver­(I) helicate complex can serve as an effective electrochromic material and can be used in electrochromic devices. It was also shown that the presence of Ag­(I) ions facilitates the formation of a thin layer of polymer on the electrode surface and improves the electrochromic performance of the polymer.

## Graphical abstract

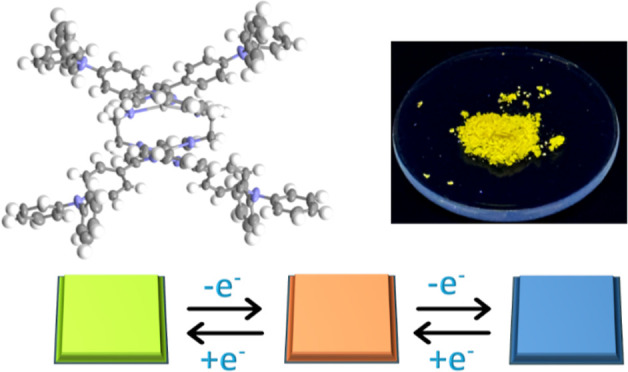

## Introduction

Supramolecular chemistry is a chemistry of architectures formed using different types of noncovalent interactions and it is one of the most investigated areas of modern chemistry in recent years.^1–3^ In comparison to traditional covalent chemistry, supramolecular systems rely on interactions that are weaker than covalent bonds, such as hydrogen bonding,^4^ metallophilic^5^ and van der Waals forces,^6^ metal coordination,^7^ or π–π stacking.^8^ The supramolecular chemistry approach enables the design and formation of complex structures with a variety of functionalities.^9–11^

An interesting example of transition metal assemblies is helicate complexes. The term ‘helicate’ was introduced by Jean Marie-Lehn in 1987 and describe an architecture of a helix coiled around the same (common) axis.^12^ The twist can appear through the coordination of a polydentate organic ligand and metal ion. Their structural ability to encapsulate guest molecules renders them attractive candidates as a MOF.^13^ Moreover, transition metal helicates arouse the interest of scientists due to their tunable magnetic,^14,15^ redox,^16–18^ photocatalytic,^19,20^ and optical^21–23^ properties. The formation of the helical structure can be influenced by many factors, such as metal:ligand stoichiometry,^24^ relative size of anion or cation,^25^ type of solvent,^26^ hydrogen bonding,^27^ and steric hindrance.^28^ These factors collectively influence the formation and stability of the helical structure, resulting in the formation of a well-defined helical-type complex with a chiral or pseudochiral helical arrangement. This structural phenomenon has implications for the complex’s properties, including its luminescence and stability.

Silver­(I) ions are very often used in the formation of different supramolecular assemblies. They are also known to form argentophilic interactions, which have been studied as an interesting factor that can be used in the construction of supramolecular systems.^29–32^ Silver­(I) complexes exhibit specific physicochemical properties that make them interesting materials for various applications, but the suitability of Ag­(I) complexes for electrochromic applications has not been investigated. Ag­(I) ions can only be reduced to metallic Ag^33,34^ and do not undergo reversible oxidation/reduction processes, which limits their electrochromic applications, but the connection of Ag­(I) ions with electroactive ligand molecules may result in formation of interesting materials for electrochromic applications. One of the interesting electroactive groups is triphenylamine. The key to triphenylamine’s electroactivity lies in its conjugated structure and electron-donating nitrogen center. Triphenylamine is recognized for its ability to participate in redox processes, which is critical for developing electrochromic and luminescent materials.^35^ Its nonplanar structure creates a three-dimensional network that is beneficial for charge transfer, making it ideal for applications in organic electronics^36,37^ and optoelectronics.^38,39^

Herein, a silver­(I) helicate obtained by the self-assembly of the N_4_-donor organic ligand and Ag­(I) ions is presented. The ligand has electroactive triphenylamine groups that enable the formation of polymers by electropolymerization. The electrochemical and luminescent properties of the Ag­(I) complex were investigated. The Ag­(I) polymer was also found to exhibit interesting electrochromic properties. It was found that the presence of Ag­(I) ions facilitates electropolymerization and improves the electrochromic properties of the polymer.

## Results and Discussion

### Synthesis

The N_4_-donor ligand **L** containing triphenylamine groups was prepared in a two-step synthesis as outlined in Figure 1.

**Figure 1:**
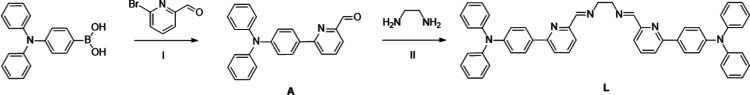
Synthesis scheme for the preparation of ligand **L**. I. K_2_CO_3_ (3eq), TBABr, Pd­(PPh_3_)_4_ (5% mol), toluene/water (2:1 v/v) 24 h, reflux; II. EtOH abs., 48 h, reflux.

The first step of the synthesis involved the Suzuki–Miyaura coupling reaction between 4-(diphenylamino)­phenylboronic acid and 6-bromopicolinaldehyde. The reaction was carried out in similar conditions as described before^40^ in the presence of potassium carbonate as a base, tetrabutylammonium bromide as a phase-transfer catalyst, and tetrakis­(triphenylphosphine)­palladium(0) as a catalyst. 6-(4-(Diphenylamino)­phenyl)­picolinaldehyde **A**, obtained in this way, was reacted with ethylenediamine in the next step of the synthesis. As a result of this condensation reaction, ligand **L** was obtained. The reaction was conducted for 48 h at reflux using absolute ethanol as a solvent. The solution was concentrated, and the final product was precipitated when put into an ice bath, washed with cold ethanol, and dried. Ligand **L** has been obtained as a white powder (yield: 89%).

Ligand **L** was further subjected to a complexation reaction with silver­(I) nitrate. Silver­(I) ions have been chosen for the reaction due to their known ability to adopt tetrahedral coordination geometry and formation of helical or polymeric complexes with multitopic ligands.^32,41,42^ The mixture of dichloromethane and acetonitrile (1:1 v/v) was chosen as a solvent to ensure the solubility of both organic ligand and metal salt. The complexation reaction was carried out in a 1:1 metal to ligand molar ratio for 24 h, at room temperature. Afterward, the solution was concentrated, and diethyl ether was added. This resulted in crystallization of greenish-yellow crystals of the Ag­(I) complex. The precipitated crystals were centrifuged and and washed with diethyl ether. The mass spectra of the compounds were recorded using ESI-MS in positive ion mode. The peak observed at *m*/*z* 725 has been assigned to the protonated ligand molecule [L + H]^+^, while the peak at *m*/*z* 393 corresponds to the dication [**L** + Na + K]^2+^. In the case of the Ag­(I) complex, the peak at *m*/*z* 833 was observed, and it has been attributed to the [**Ag**_**2**_ **L**_**2**_ + 2H]^2+^ dication. This confirms the formation of the complex of a 2:2 metal to ligand stoichiometry. The ESI-MS spectra are shown in Figure S10A,B. To investigate the structure of the obtained complex, a single crystal suitable for X-ray analysis was obtained by the slow diffusion method. The crystallographic analysis confirmed that the complex is a double-stranded helicate as shown in Figure 2.

**Figure 2:**
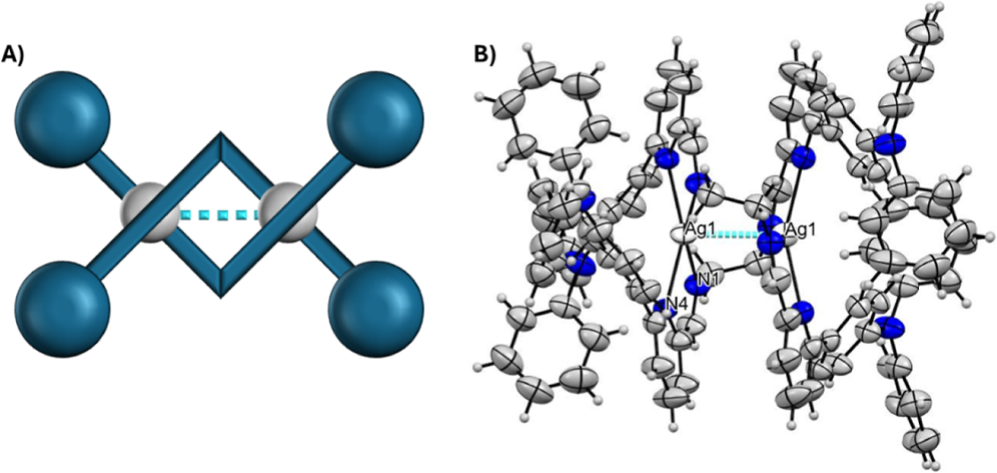
(A) Schematic representation of the **Ag**_**2**_ **L**_**2**_ complex; (B) anisotropic-ellipsoid representation of the **Ag**_**2**_ **L**_**2**_ complex. (Ag–Ag interactions are shown in light blue).

The **Ag**_**2**_ **L**_**2**_ complex is highly *D*_*2*_-symmetric; the asymmetric part of the unit cell consists of two-halves of Ag atoms (which lie on the 2-fold axis along *z*) and one-half of the ligand (*Z*′ = 1/4). The Ag centers are four-coordinated in a N_4_ manner (Table 1), and each ligand molecule gives two nitrogen atoms to each Ag center, in a geometry which could be described as a highly distorted square planar one (Figure 2).

**Table 1:** Relevant Geometric Parameters (Å, °) with Their Standard Uncertainties (s.u.’s) in Parentheses^a^

Ag1–N1	2.279(2)
Ag1–N4	2.499(2)
N1–Ag1–N1^i^	158.64(11)
N4–Ag1–N4^i^	154.98(11)
N1–Ag1–N4	71.34(7)
Ag1–Ag1^ii^	3.0474(6)

a Symmetry codes: ^i^1 – *x*, 3/2 – *y*,*z*; ^ii^1 – *x*, *y*,3/2 – *z*.

The overall coordination of the complex—thanks to the flexibility of the ligand—allows for the short Ag–Ag contact of 3.0474(6) Å—such contacts are often observed in similarly constrained complexes. For example, an average distance between two Ag­(I) ions of 2.96 Å was found in poly-NHC-based tetranuclear silver helicate.^43^ Shorter Ag–Ag distances of 2.8–2.9 Å were observed for Ag­(I) complexes with a Schiff base type ligand.^44^

In the case of helicates obtained from nonchiral ligands, it is possible for the complexes to crystallize in two forms as a “racemic mixture” when the individual crystals contain only one enantiomer or as a “racemic compound” when both enantiomers are present in one crystal lattice.^45,46^ In fact, the **Ag**_**2**_ **L**_**2**_ complex crystallizes in a centrosymmetric space group, so it is a racemic mixture. Figure 3 shows the crystal packing of the complex, as viewed down the *a* axis. The molecules of the Ag­(I) complex form homochiral layers that are parallel to the *b* axis and layers of alternating chirality lie side by side in sheets parallel to the *ab* plane.

**Figure 3:**
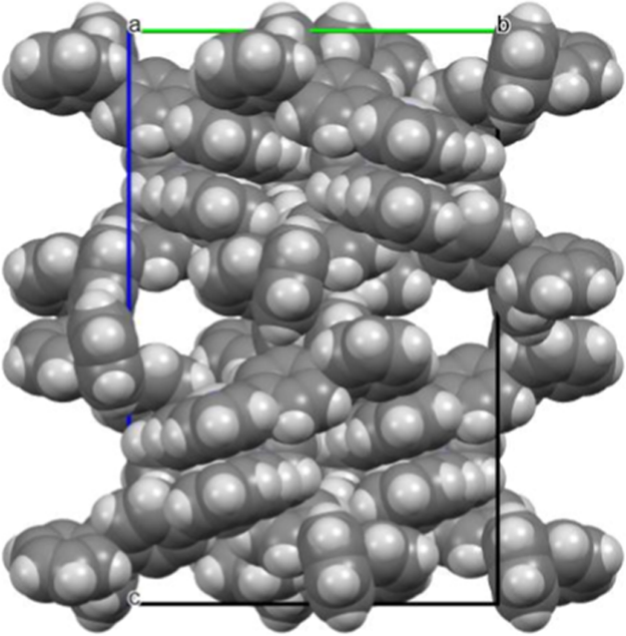
Partial view of cation layers found in the Ag­(I) complex.

### Photophysical Properties

In the next step, the photophysical properties of both the ligand and Ag­(I) complex were investigated. This was done to evaluate the usefulness of the materials for optoelectronic applications. First, the absorption and emission properties of obtained compounds were investigated in dichloromethane solutions at concentrations of 2 × 10^–5^ M (**L**) and 1 × 10^–5^ M (Ag­(I) complex). Such a concentration was chosen to maintain the same molar amount of ligand molecules in solution. The absorption and emission spectra of the ligand and Ag­(I) complex are shown in Figures S1 and S2, and photophysical data are collected in Table 2.

**Table 2:** Photophysical Properties of Ligand **L** and the **Ag­(I)** Complex in Dichloromethane Solutions

compound	λ_abs_ (nm)	λ_em_ (nm)	Stokes shift (cm^–1^)	$\varepsilon \left(\frac{dm^{3}}{mol\times cm}\right)$
ligand **L**	230			
	350	442	5947	1.88 × 10^5^
**Ag(I)** complex	229			
	295			
	350	437	5688	1.68 × 10^5^

It was observed that the complexation with Ag­(I) ions does not affect the photophysical properties of the ligand molecules. The absorption maxima were at 230 and 350 nm for both the ligand and Ag­(I) complex. This was ascribed to π–π* and *n*–π* transitions originating from the triphenylamine groups and pyridyl rings of ligand **L**.^32,47,48^ Additionally, the complex exhibited an absorption band with the maximum at 295 nm, which can be attributed to the transition originating from intramolecular Ag–Ag interactions.^49,50^ The emission of the Ag­(I) complex was only slightly shifted from 442 nm for ligand **L** to 437 nm for the Ag­(I) complex. Additionally, for the Ag­(I) complex, the shoulder at 520–700 nm was observed when excited at 295 nm. This can be assigned to ligand-to-metal charge transfer transition (LMCT) modified significantly by the presence of the Ag–Ag interactions, designated as ligand-to-metal–metal charge transfer (LMMCT).^29^

The emission properties of the ligand **L** and its Ag­(I) complex in the solid state were also investigated. Figure 4 shows the excitation and emission spectra of **L** and the Ag­(I) complex. The ligand **L**, when excited at its absorption maximum, exhibited emission in the blue region of the spectrum with the maximum centered at 407 nm. In comparison, the Ag­(I) complex exhibited a sharp emission band at 462 nm and broad weak emission spreading from 475 to 700 nm. The higher energy emission band was found to be similar in shape with the emission band of ligand **L**, which suggests that the origin of this emission band is derived from ligand-centered transitions, although modified by metal ions.^29,51,52^ In turn, the broad emission band may be assigned to ligand-to-metal charge transfer transition perturbated by the presence of argentophilic interactions.^29^ This is consistent with emission properties of other polynuclear Ag­(I) complexes in which Ag–Ag interactions occur.^53,54^

**Figure 4:**
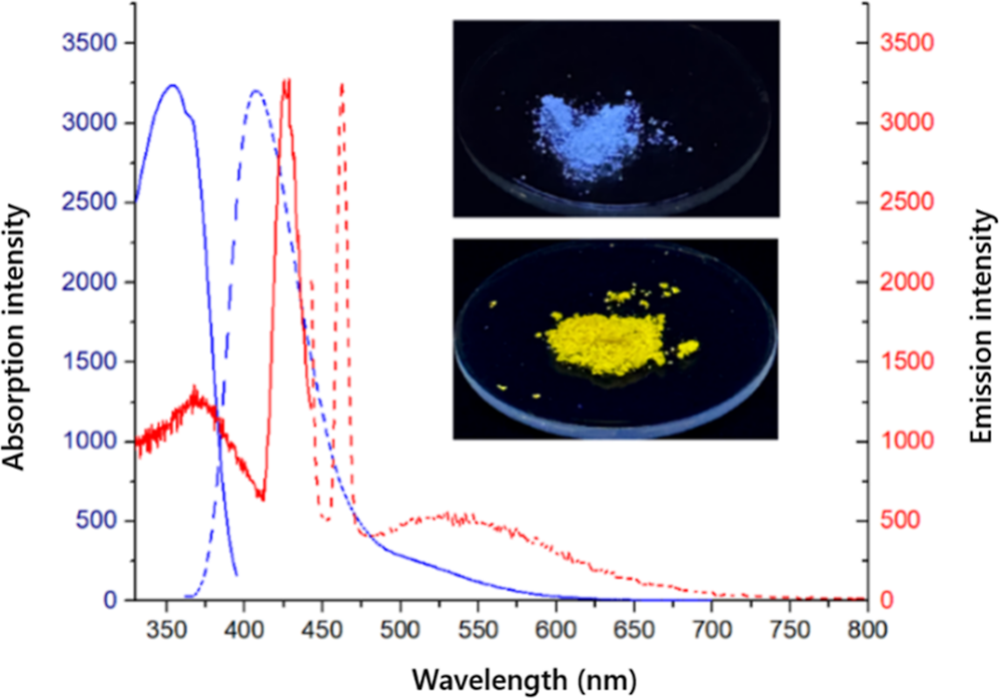
Excitation (solid line) and emission (dashed line) spectra of ligand **L** (blue) and the **Ag­(I)** complex (red) measured in the solid state. Insert: photographs showing the emission of ligand **L** (top) and the Ag­(I) complex (bottom) when excited using an UV lamp (365 nm).

As seen in photographs inserted in Figure 4, the ligand when excited with the UV lamp (365 nm) exhibited an emission of blue light, while the complex emitted yellow light. The excitation maximum of ligand **L** was observed to be at 354 nm when the emission was monitored at 406 nm, while for the complex, the excitation maximum was red-shifted to 428 nm (emission was monitored at 462 nm).

### Electrochemical Properties

Electrochemical properties of ligand **L** and its Ag­(I) complex were investigated by cyclic voltammetry using the platinum working electrode (diameter ϕ = 2 mm), platinum wire, and nonaqueous Ag/Ag^+^ electrode calibrated using ferrocene as an external standard as a counter and reference electrode, respectively. The degassed and anhydrous 0.1 M solution of tetrabutylammonium hexafluorophosphate (TBAPF_6_) in dichloromethane was used as a supporting electrolyte. To investigate the electropolymerization ability of both the ligand and Ag­(I) complex, multiple oxidation/reduction cycles were recorded. The cyclic voltammograms were recorded at concentrations of 4 × 10^–4^ M for the ligand and 2 × 10^–4^ for the complex. 60 or 40 successive redox cycles for the ligand and Ag­(I) complex, respectively, were performed to obtain thin layers of polymers. As shown in Figure 5A,B, the gradual increase in the current was observed with each redox cycle, indicating that the polymers were deposited on the electrode surface. It was found that Ag­(I) complex electropolymerizes more easily compared to ligand **L**. As it is known, the electropolymerization ability of triphenylamine derivatives depends largely on the molecular structure and the amount of charge at the 4′ position of the triphenylamine radical cation.^55^ Therefore, it can be assumed that the presence of Ag­(I) ions, due to their electron-accepting properties, which may be enhanced by the presence of argentophilic interactions^56^ changes the electron distribution, facilitating the electropolymerization. The deposition of the polymer on the electrode surface occurred due to the formation of carbon–carbon bonds between two triphenylamine groups, which leads to obtaining the tetraphenylbenzidine (TPB) group.^57–59^ The dimerization via tail-to-tail coupling at the *para* positions of the phenyl rings is a common reaction pathway in the electrochemical oxidation of triphenylamines, because *ortho*-coupling can occur under specific conditions only, particularly when the *para* positions are blocked or when directing groups favor *ortho*-selective C–H amination.^60,61^ After electropolymerization, the electrodes were removed from the electrolyte solution and washed with dichloromethane, and the electrochemical behavior of polymers was investigated in the monomer-free electrolyte. It was found that the **poly-L** exhibited major oxidation/reduction waves at *E*_p,a_ = +592 mV and *E*_p,c_ = +541 mV with minor shoulders at +415 mV and +357 mV, respectively, while in the case of **poly-Ag**, two successive oxidation/reduction couples were observed at E_p,a1_ = +605 mV, *E*_p,a2_ = +426 mV and *E*_p,c1_ = +528 mV, *E*_p,c2_ = +370 mV (Figure S3). These redox processes are typical for compounds containing two triphenylamine groups and are connected with the formation of a radical cation on a triphenylamine group in its first oxidation process.^40,59,62^ The second oxidation process comes from oxidizing the radical cation to a dications form as closed-shell singlets^63,64^ or diradical dications reported as triplets or open-shell singlets.^65,66^ For **poly-L**, these oxidation/reduction couples overlap and become almost undistinguishable in the cyclic voltammogram.^67,68^ This may indicate that the presence of Ag­(I) ions influences the electrochemical properties of the complex, stabilizing intermediate oxidation states.^69^

**Figure 5:**
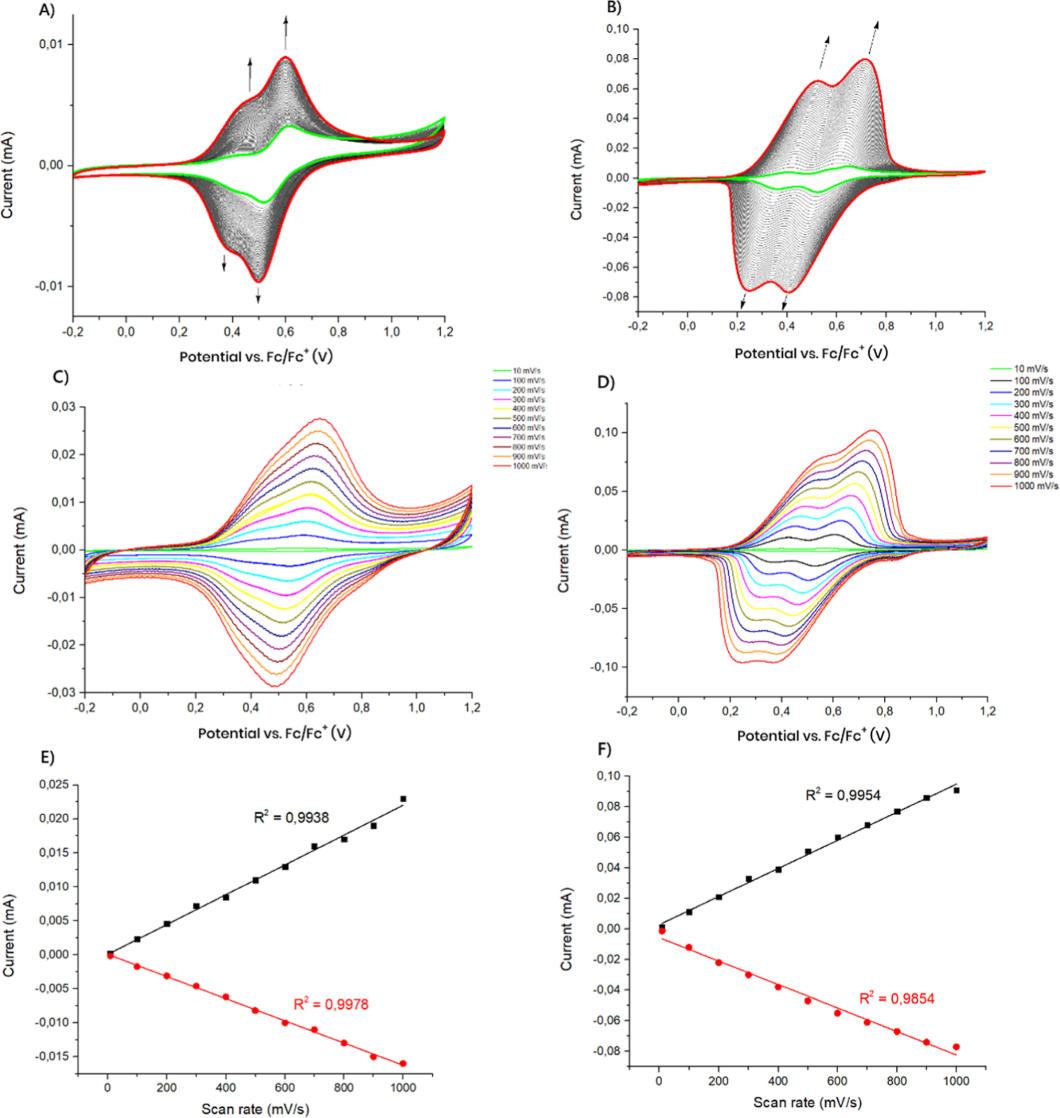
(A) Electropolymerization of ligand **L** on the Pt working electrode by 60 repeated CVs at a scan rate of 100 mV/s, green—first cycle, red–last cycle; (B) electropolymerization of the **Ag**_**2**_ **L**_**2**_ complex on the Pt working electrode by 40 repeated CVs at a scan rate of 100 mV/s; CVs of **poly-L** (C) and **poly-Ag** (D) obtained at scan rates from 10 to 1000 mV/s; dependence of the anodic and cathodic peak currents as a function of scan speed for **poly-L** (E) and **poly-Ag** (F).

No prewave was observed during the electrochemical measurements, which may indicate that ion transport channels of sufficient size are inherently formed within the polymer matrix during the electropolymerization process. This observation suggests that minimal structural reorganization of the polymer is required to support efficient electron transfer throughout the film with negligible polymer “breathing” or dynamic swelling involved.

The electrochemical properties of formed polymers were further investigated in a monomer-free electrolyte by the recording of cyclic voltammograms of polymers differing in scan rates (from 10 to 1000 mV/s) (Figure 5C,D). It was found that as the scan rate increases, both anodic and cathodic peak currents (*I*_p_) scale proportionally with the scan rate (*v*). This suggests that electrochemical processes are confined on an electrode surface. The linear regression coefficients (*R* ^2^) were calculated to be 0.9938 and 0.9978 (**poly-L**) and 0.9954, and 0.9854 (**poly-Ag**) for the anodic and cathodic current, respectively (Figure 5E,F).

### Polymer Characterization

To investigate the chemical composition of the polymer to confirm the presence and oxidation state of Ag­(I) ions in the obtained polymer, X-ray photoelectron spectroscopy (XPS) was carried out. XPS is a crucial technique for analyzing the elemental composition, oxidation states, and chemical environment of metalloorganic polymeric films, providing information about their surface chemistry and coordination structure. The XPS survey spectra of the Ag­(I) complex and **poly-Ag** are shown in Figures S4 and S5. As can be seen, both spectra contain peaks of the C 1s, N 1s, and Ag 3d core levels. Additionally, the **poly-Ag** spectrum contains signals from Si and O atoms from the substrate, as well as F 1s and P 2p core levels from the PF_6_^–^ anions of the electrolyte. The Ag 3d signal can be deconvoluted into two spin–orbit peaks (Figures 6 and S6), which are observed at the binding energies of 368.5 eV (Ag 3d_5/2_) and 374.5 eV (Ag 3d_3/2_) for **poly-Ag**, as well as 368.2 and 374.2 eV for the Ag­(I) complex. This is consistent with the +I oxidation state of silver ions.^70^

**Figure 6:**
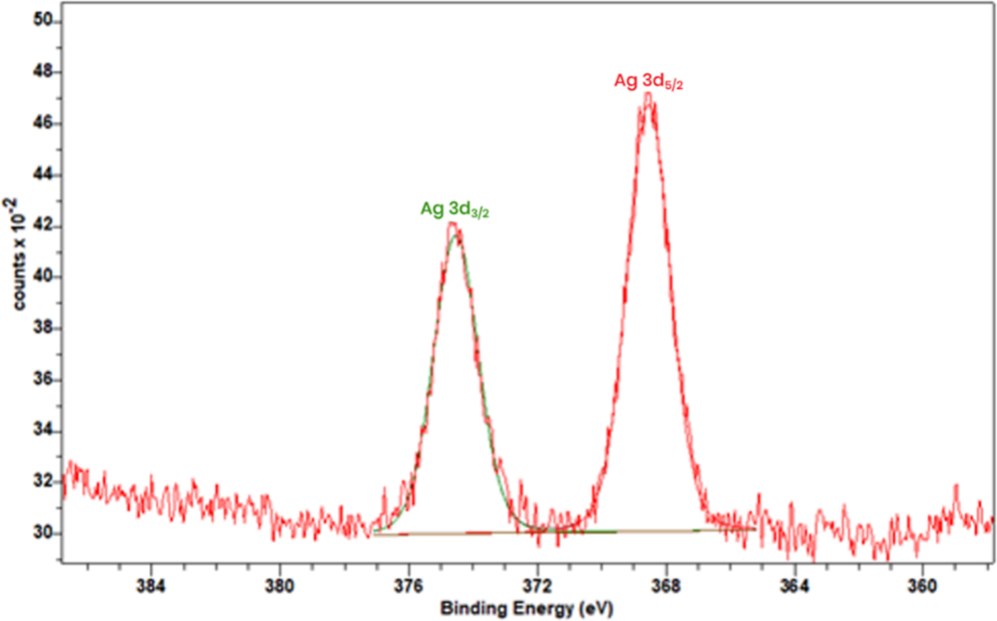
High-resolution XPS spectra of Ag 3d core levels of the **poly-Ag** sample.

The morphology of the layer was further investigated by scanning electron microscopy (SEM) (Figure 7).

**Figure 7:**
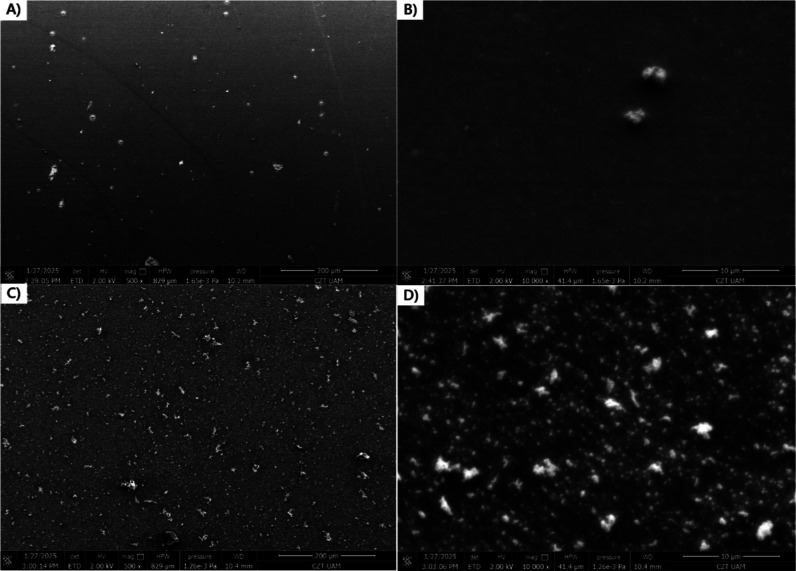
SEM images of thin films of ligand (A,B) and Ag­(I) complex (C,D) polymers with magnification 500× (A,C) and 10,000× (B,D).

The polymer layer of **poly-L** exhibits a relatively smooth and homogeneous surface with minor irregularities, suggesting uniform film formation. In contrast, the polymeric Ag­(I) complex film presents an unsmooth surface with a more granular morphology containing numerous particles and some aggregates. Because the helicate presents stiff, organized supramolecular architecture, it probably promotes uniform packing and controls film growth during electropolymerization, leading to obtaining more three-dimensional film compared to the ligand.^71^

The thickness of the polymer layers was investigated by using atomic force microscopy (AFM). For this, the polymers were cut with a blade and their thickness was measured as a difference between bare ITO and the polymer surface. Figure S7C,D shows the step-high differences in **poly-L** and **poly-Ag**, respectively. The profiles were investigated in four different places, as shown in Figure S7A,B. It was measured that the thickness of **poly-Ag** was ∼450 nm, while **poly-L** was much thinner (∼50 nm), although a higher number of oxidation/reduction cycles were performed to electropolymerize **poly-L**. This further suggests that the presence of Ag­(I) ions enhances the electropolymerization process.

Obtained polymeric films were further investigated for their wettability (Figure 8). Contact angle measurements allow us to determine how an electrochromic material interacts with liquids. This is crucial for understanding how the material will respond to moisture and can provide insights into the interactions of the material with liquids, which is important for applications like smart windows and flexible electronics.^72,73^ The contact angle measurements were evaluated by using the sessile drop research method at 23 °C, with deionized water as the probe liquid. The obtained contact angle values showed the moderate hydrophobicity of both polymer materials. The polymer film derived from the free ligand exhibited an average water contact angle of 69.7°, whereas the polymeric film of the Ag­(I) complex showed a slightly lower contact angle of 66.6°. This suggests that the presence of Ag­(I) does not significantly change the hydrophobicity of the material. This may be because metal ions are surrounded by ligand molecules, forming a helicate.

**Figure 8:**
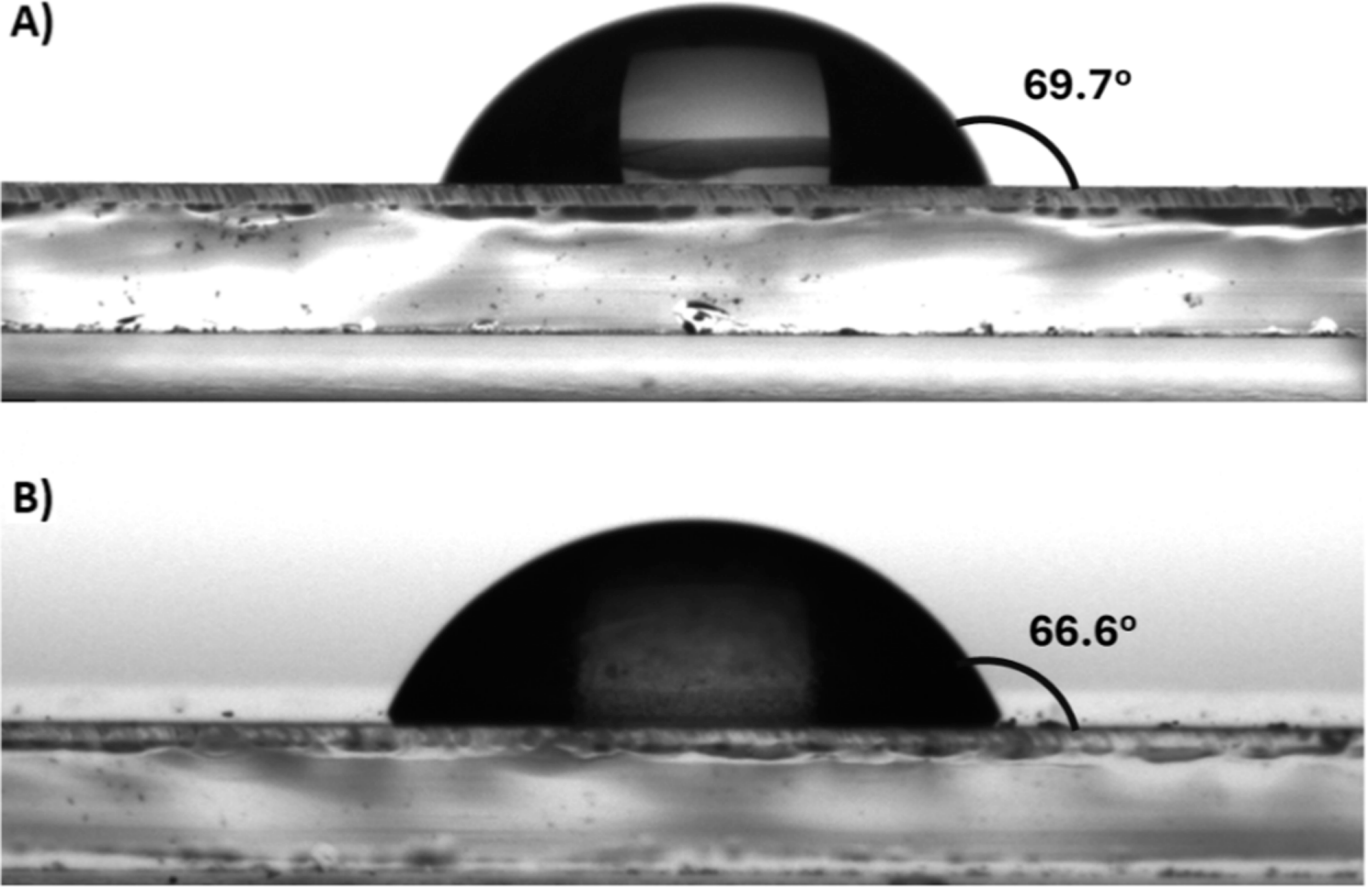
Water contact angles of **poly-L** (A) and **poly-Ag** (B).

### Electrochromic Properties

To investigate the usefulness of obtained polymers as an electrochromic material, spectroelectrochemical studies were performed, and it is shown in Figures 9 and S8. It was found that ligands **poly-L** and **poly-Ag** undergo reversible color changes with the applied potential. **Poly-Ag** in its neutral state shows one absorption band with the maxima at 369 nm (Figure 9). With increase in electric potential up to +0.6 V and oxidation of the polymer, the intensity of this absorption band slowly decreased and a simultaneous appearance of two new absorption bands, one in the visible region with the maxima at 490 nm and the second broad band in the NIR region at ∼1400 nm, was observed. This was connected with the formation of the radical cation on the TPB group, and the absorption band in the NIR region was ascribed to intervalence charge transfer (IVCT) transition between the two TPA groups in the TPB moiety being in two different redox states: neutral and radical cation as observed for similar systems.^74,75^ This resulted in a color transition from yellow to orange. A clear isosbestic point at 422 nm for this step was observed, indicating that the TPB → TPB^•+^ redox process occurs without any side reactions. The further increase of the potential up to +1.0 V caused the color to change to blue. The absorption bands characteristic for the TPB radical cation disappeared and the broad absorption band centered at 759 nm appeared and it can be assigned to transition TPB^•+^ → TPB^2+^ and formation of the dication.

**Figure 9:**
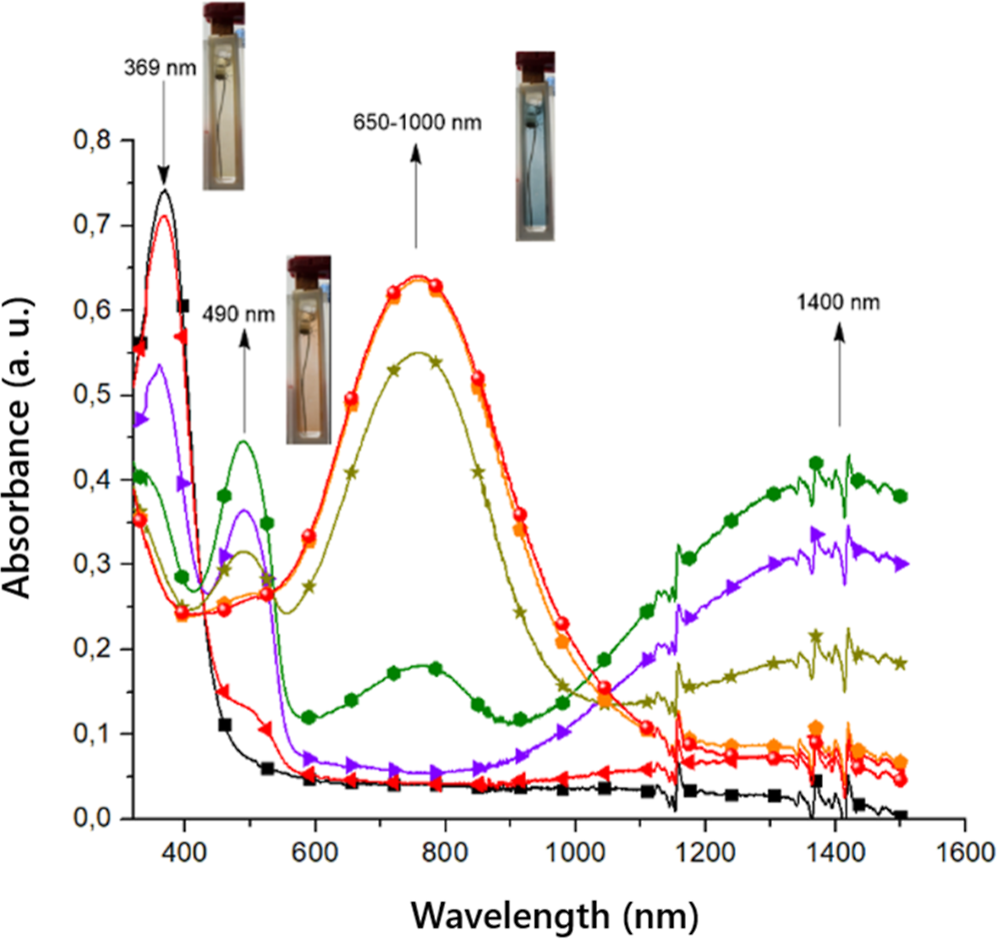
Spectroelectrochemistry of **poly-Ag** measured in an anhydrous and deaerated 0.1 M solution of TBAPF_6_ in dichloromethane as a supporting electrolyte versus Fc/Fc^+^ with photographs of the appropriate polymer oxidized state. Applied potentials: 0 V (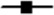), 0.3 V (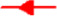), 0.4 V (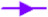), 0.5 V (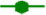), 0.6 V (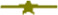), 0.7 V (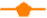), and 0.8 V (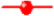).

On the other hand, **poly-L** undergoes only one color transition (Figure S8). The neutral state of the polymer is characterized by the absorption band with the maximum at 343 nm, blue-shifted compared to **poly-Ag**. The electrooxidation of the polymer resulted in the disappearance of this band, and the formation of a broad absorption band spreading from the visible to the NIR region at 400–1100 nm was observed. This can be attributed to the formation of a radical cation and the dication of TPB triphenylamines at the same time. This observation is consistent with the electrochemical data obtained for this polymer. The color change was from yellow to blue and the color intensity was found to be much lower compared to **poly-Ag**. This relates to the fact that the layer of **poly-L** was much thinner compared to that of **poly-Ag**. For both polymers, applying a potential of 0 V led to the neutralization of the oxidation states and the recovery of spectra characteristic of the neutral state, which proves that the oxidation processes are reversible.

The electrochromic stability of **poly-L** and **poly-Ag** was evaluated using UV–vis–NIR spectroscopy coupled with the chronoamperometry method, monitoring transmittance at 800 and 760 nm, for **poly-L** and **poly-Ag**, respectively (Figure S9A,B). The measurements were done using 0.1 M LiClO_4_ in dichloromethane:acetonitrile (1:1 v/v) as a supporting electrolyte. For **poly-L**, a gradual decrease in the transmittance difference (Δ*T* %) was observed—from 63.7% to 26.6% after 84 oxidation/reduction cycles (Figure S9A). This suggests the degradation of the polymer layer over time. In contrast, **poly-Ag** demonstrates improved long-term stability, maintaining its electrochromic response over an extended period of 9000 s (300 oxidation/reduction cycles) (Figure S9B). The transmittance difference decreased from the initial 77.6% to 19.8%. This improved stability of **poly-Ag** compared to **poly-L** is probably due to the higher degree of cross-linking of this polymer^76^ because metal atoms act as cross-linking agents that create a three-dimensional network.^77^ A similar effect was previously observed for metallopolymer containing Fe­(II) ions.^40^ Additionally, the presence of metal ions in the polymer, due to their electron-accepting properties, leads to the formation of a donor–acceptor (D–A) structure, and this type of polymer is characterized by better performance compared to non-D–A polymers.^78,79^ The enhanced durability of the electrochromic properties of **poly-Ag**, compared to **poly-L**, made it a more suitable candidate for further investigation as an electrochromic material.

The electrochromic properties of **poly-Ag** including switching time (coloration and bleaching) were further investigated using double-potential step chronoamperometry coupled with an UV–vis–NIR spectrometer. The response time (*t*_90%_) refers to the time required for the material to reach 90% of its total optical change (coloration or bleaching) upon application of an electric potential. The coloration and bleaching time of **poly-Ag** were calculated to be 2.7 and 2.8 s, respectively (Figure 10A). The changes in the transmittance difference for **poly-Ag** were further investigated by switching potential between 1.2 and 0 V in time intervals of 40, 30, 20, 10, and 5 s (Figure 10B). This was done to evaluate the effect of the retention time on the contrast ratio of the material. It has been observed that the transmittance difference does not change significantly along with the time intervals of potential switching. This is consistent with the fast switching times calculated for the polymer.

**Figure 10:**
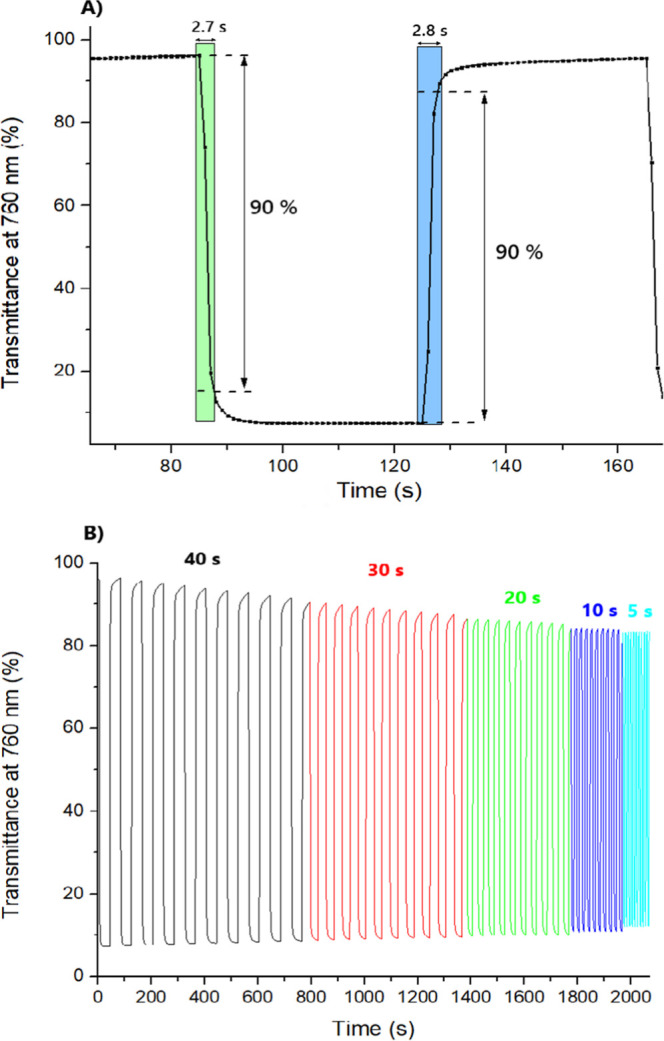
(A) Coloration and bleaching times of the Ag­(I) polymeric complex; (B) the difference in transmittance monitored at 760 nm between the oxidized form (1.2 V) of the polymeric complex and its neutral form (0 V) measured with decreasing width of voltage pulses from 40 to 5 s.

## Experimental Section

### Materials and Methods

All reagents and solvents were obtained from commercial suppliers (Aldrich and Fluorochem) and used without further purification. Compound **A** was obtained in a similar way as described before.^40^ NMR spectra were run on a Bruker 600 MHz-AdvanceNEO spectrometer and were calibrated against the residual protonated solvent signals (DMSO 2.50 ppm), and shifts are given in parts per million. Mass spectrometry analysis was conducted on a QTOF mass spectrometer (Impact HD Bruker, Bruker Daltonics, Bremen, Germany). Absorption measurements were performed by using an UV–vis–NIR Jasco V-770 spectrometer. Emission and excitation spectra were recorded at room temperature in dichloromethane solution using a Jasco FP-8500 spectrometer (Jasco, Japan), and the solutions were degassed by bubbling argon for 15 min. The emission and excitation spectra of ligand **L** and the Ag­(I) complex in the solid state were obtained using a 100 mm integrating sphere, which had been calibrated with a calibrated light source. SEM images were acquired on a scanning electron microscope (Quanta 250FEG), and AFM images were obtained using an Agilent 5500 atomic force microscope. XPS spectra were measured using Specs UHV/XPS/SPM using Al Kα as an X-ray source. The spectra were analyzed using CasaXPS software, and the binding energies were standardized using the C 1s peak at 285.0 eV. Goniometric measurements were prepared using goniometer Krüss DSA100E with software Krüss DSA 4 (drop shape analyzer). Electrochemical measurements were done using a multichannel BioLogic VSP potentiostat using an anhydrous 0.1 M solution of tetrabutylammonium hexafluorophosphate (TBAPF_6_) in dichloromethane (DCM) or lithium perchlorate (LiClO_4_) in a dichloromethane:acetonitrile mixture (1:1 *v*/*v*) as a supporting electrolyte. A platinum electrode was used as a working electrode, platinum wire as an auxiliary electrode, and a nonaqueous Ag/Ag^+^ electrode as a reference electrode. Spectroelectrochemical measurements were done using a multichannel BioLogic VSP potentiostat connected to a Jasco V-770 UV–vis–NIR spectrometer. An ITO plate was used as a working electrode with a platinum wire as an auxiliary electrode and a nonaqueous Ag/Ag^+^ electrode as a reference electrode. The Ag/Ag^+^ electrode was assembled by immersing a clean silver wire in a glass capillary with a porous tip containing the solution of 0.01 M AgNO_3_ and 0.1 M TBAP in acetonitrile as the supporting electrolyte. Before polymerization, ITO plates were cleaned by sonication in water for 15 min, followed by sonication in 2-propanol for 15 min, dried, and cleaned by ozone generation using the Ossilla UV–ozone cleaner. The reference electrode was calibrated using ferrocene prior to use and obtained oxidation/reduction potentials were recalculated vs a Fc/Fc^+^ redox couple (*E*_1/2Fc/Fc^+^_ = 0 V). The ligand and Ag­(I) complex were electropolymerized on ITO electrodes to coat them with **poly-L** and **poly-Ag**. These were used for SEM and AFM imaging.

### Crystal Structure Determination

Diffraction data were collected by the ω- and ϕ-scan technique, at room temperature, using graphite-monochromated MoKα radiation (λ = 0.71073 Å), on a Bruker D8 Quest four-circle diffractometer. The data were corrected for Lorentz polarization as well as for absorption effects.^80^ Precise unit-cell parameters were determined by a least-squares fit of the reflections of the highest intensity chosen from the whole experiment. The structures were solved with SHELXT^81^ and refined with the full-matrix least-squares procedure on F^2^ by SHELXL.^82^ All non-hydrogen atoms were refined anisotropically. Hydrogen atoms were placed in idealized positions and refined as ‘riding model’ with isotropic displacement parameters set at 1.2 (1.5 for methyl groups) times U_eq_ of appropriate carrier atoms.

#### Crystal Data

C_100_H_80_Ag_2_N_12_, *M*_r_ = 1665.50, orthorhombic, *Ccca*, *a* = 17.9872(10) Å, *b* = 19.1844(10) Å, *c* = 29.8431(17) Å, *V* = 10298.1(10) Å^3^, *Z* = 4, *d*_*x*_ = 1.074 g cm^–3^, *F*(000) = 3432, μ = 0.426 cm^–1^, 64,341 reflections collected, 4716 symmetry independent (*R*_int_ = 6.17%), 3716 with *I* > 2σ­(*I*). Final *R*[*I* > 2σ­(*I*)] = 0.0403, w*R*2­[*I* > 2σ­(*I*)] = 0.1073, *R* [all reflections] = 0.0514, w*R*2­[all reflections] = 0.1144, S = 1.043, (Δρ_max_/Δρ_min_) = 0.30/–0.34 e Å^–3^. CCDC deposition number: 2427267.

### Synthetic Procedures

#### Ligand **L**

718 mg (2.05 mmol) of compound **A** was dissolved in absolute ethanol (30 mL). To a stirring solution, 68 μL (1.025 mmol) of ethylenediamine was added dropwise. The reaction was carried out at 70 °C. After 48 h, the reaction mixture was concentrated, and a white solid was precipitated via a chilling process in the ice bath. The final product was washed with cold ethanol and dried. White solid, 645 mg (yield 89%). ^1^H NMR (600 MHz, DMSO) δ: 8.40 (s, 1H), 7.97 (d, *J* = 8.9 Hz, 2H), 7.91–7.85 (m, 2H), 7.82 (dd, *J* = 6.7, 2.1 Hz, 1H), 7.37–7.30 (m, 4H), 7.09­(tt, *J* = 7.5, 1.2 Hz, 2H), 7.08–7.05 (m, 4H), 7.03–6.98 (m, 2H), 4.01 (s, 2H) ppm. ^13^C NMR (151 MHz, DMSO) δ: 163.4, 155.5, 153.8, 148.3, 146.8, 137.8, 131.6, 129.7, 127.7, 124.6, 123.6, 122.2, 120.7, 118.4, 60.5 ppm. ESI-MS *m*/*z* = 725 (10%) (L + H)^+^, 393 (100%) (**L** + Na + K)^2+^.

#### Complex **Ag**_**2**_ **L**_**2**_

Thirty milligrams (0.04 mmol) of ligand **L** were dissolved in acetonitrile:dichloromethane 1:1 *v*/*v* (3 mL:3 mL) and 7.03 mg (0.04 mmol) of silver­(I) nitrate was added afterward. The reaction was carried out overnight at room temperature under a normal atmosphere. The final complex was obtained as green shining crystals after concentrating and adding a small amount of diethyl ether followed by centrifuging and washing with diethyl ether. Green crystals, 28 mg (yield 82%) ^1^H NMR (600 MHz, DMSO) δ: 8.36–8.32 (m, 1H), 7.95 (t, *J* = 7.7 Hz, 1H), 7.84–7.79 (m, 1H), 7.47 (d, *J* = 8.2 Hz, 2H), 7.40 (d, *J* = 7.5 Hz, 1H), 7.36–7.29 (m, 4H), 7.11 (tt, *J* = 7.4, 1.2 Hz, 2H), 6.86 (d, *J* = 7.8 Hz, 4H), 6.50 (d, *J* = 8.2 Hz, 2H), 3.99 (s, 1H), 3.57 (s, 1H) ppm. ESI-MS *m*/*z* = 833 (100%) (**Ag**_**2**_ **L**_**2**_ + 2H)^2+^.

## Conclusions

In this work, a novel silver­(I) helicate complex containing a N_4_-donor ligand was successfully synthesized. The ligand contains two triphenylamine moieties, which are capable of electropolymerization. The complex exhibits a helicate structure as confirmed by X-ray crystallography. Both ligand **L** and the Ag­(I) complex exhibit emission in the solid state. The ligand was found to emit light in the blue range of the spectrum, while the Ag­(I) complex exhibits dual emission: the emission peak in the blue range due to the emission of ligand molecules and broad emission in the range 475 nm–700 nm due to LMCT perturbed by the presence of argentophilic interactions. Both compounds were successfully electropolymerized on the electrode surface giving electrochromic thin polymeric films. The **poly-Ag** layer exhibits a significantly higher thickness, enhanced electrochemical activity, and improved morphological features compared to **poly-L**, as well as improved electrochromic performance, characterized by multistep color changes (yellow–orange–blue) and better electrochemical stability in comparison with **poly-L**. These results highlight the potential of the silver­(I) helicate-based polymer as a promising material for applications in electrochromic and optoelectronic devices. The research also shows that the presence of Ag­(I) ions enhances electropolymerization and improves the electrochromic properties of the polymer.

## Supplementary Material



## Data Availability

The data supporting this article have been included as part of the Supporting Information.

## Notes

Additional figures including normalized absorption spectra, CVs, high-resolution XPS survey spectra, AFM micrographs, plots of layer thickness, spectroelectrochemistry, electrochromic stability, ESI-MS spectra, and ^1^H NMR and ^13^C NMR spectra (PDF)
