# Supplementary material for: Electropolymerization of Silver(I) Helicate into Conductive Metallopolymer: Structural and Functional Insights
Source: Inorg Chem. 2025 Oct 17;64(43):21387–97. doi: 10.1021/acs.inorgchem.5c02774 (PMC12587391; doi:10.1021/acs.inorgchem.5c02774)
Supplement: Supplementary file 1 [file ic5c02774_si_001.pdf]

Supplementary information

Electropolymerization of silver(I) helicate into conductive metallopolymer: structural and functional insights

Sergiusz Napierała, Maciej Kubicki, Monika Wałęsa-Chorab*

Faculty of Chemistry, Adam Mickiewicz University in Poznań, Uniwersytetu Poznańskiego 8, 61-614 Poznań, Poland

* *Correspondence:* mchorab@amu.edu.pl

Table of content

Fig. S1. Normalized absorption spectra of ligand **L** (black) and Ag(I) complex (red) measured in dichloromethane solutions at concentrations of 2 x 10^{-5} M (**L**) and 1 x 10^{-5} M (Ag(I) complex). ..3

Fig. S2. Normalized emission spectra of ligand **L** (black) and Ag(I) complex excited at 295 nm (green) and 350 nm (red) measured in dichloromethane solutions at concentrations of 2 x 10^{-5} M (**L**) and 1 x 10^{-5} M (Ag(I) complex). 3

Fig. S3. CVs of **poly-L (A)** and **poly-Ag (B)** obtained at scan rate from 100 mV/s. 4

Fig. S4. High-resolution XPS survey spectra of Ag(I) complex. 4

Fig. S5. High-resolution XPS survey spectra of **poly-Ag**. 5

Fig. S6. High-resolution XPS spectra of Ag 3d core levels for Ag(I) complex.............................5

Fig. S7. AFM micrographs of **poly-L** (A) and **poly-Ag** (B) polymer with marked locations for cross-section profile measurements. C,D) plots of layer thickness of **poly-L** and **poly-Ag**.......6

Fig. S8. Spectroelectrochemistry of **poly-L** measured in anhydrous and deaerated 0.1 M solution of $TBAPF_6$ in dichloromethane as a supporting electrolyte versus Fc/Fc^+ with photographs of appropriate polymer oxidized state. Applied potentials: 0.2 V (-■-), 0.4 V (-●-), 0.5 V (-▲-), 0.6 V (-▼-), 0.7 V (-◆-), 0.8 V (-◄-), 0.9 V (-►-), 1.0 V (-●-) and 1.1 V (-★-). 7

Fig. S9. Electrochromic stability measured between oxidized (0.8 V) and neutral (0 V) form of **poly-L** (A) and **poly-Ag** (B) measured at 800 nm and 760 nm in anhydrous and deaerated 0.1 M dichloromethane:acetonitrile (1:1 *v*/*v*) solution of $LiClO_4$ as a supporting electrolyte by switching between 30 s (A) and 15 s (B) intervals. 7

Fig. S10. ESI-MS spectra of **L** (A) and **Ag(I)** complex (B). 8

Fig. S11. ^{1}H NMR spectrum of ligand **L**. 8

Fig. S12. ^{1}H NMR spectrum of ligand **L** – expanded aromatic region. 9

Fig. S13. ^{13}C NMR spectrum of ligand **L**. 9

Fig. S14. ^{1}H NMR spectrum of Ag(I) complex – expanded aromatic region............................10

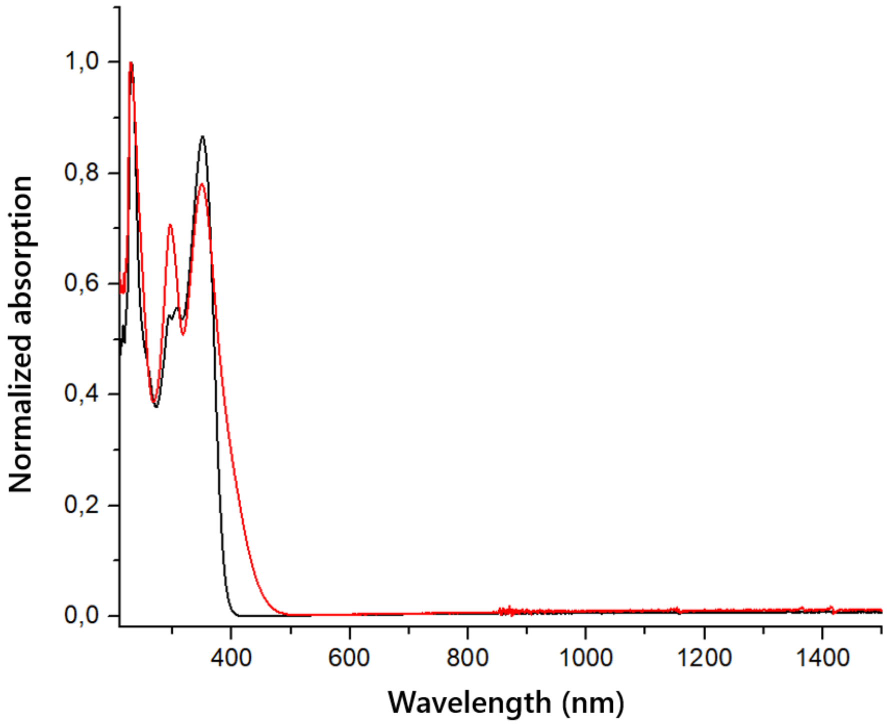

Fig. S1. Normalized absorption spectra of ligand **L** (black) and Ag(I) complex (red) measured in dichloromethane solutions at concentrations of 2 x 10^{-5} M (**L**) and 1 x 10^{-5} M (Ag(I) complex).

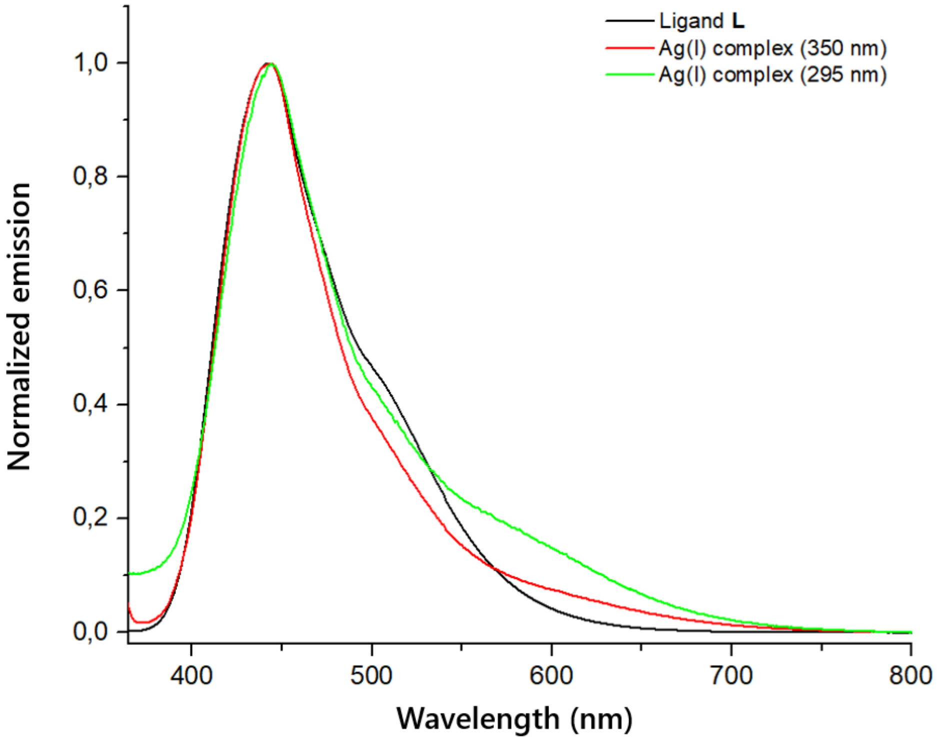

Fig. S2. Normalized emission spectra of ligand **L** (black) and Ag(I) complex excited at 295 nm (green) and 350 nm (red) measured in dichloromethane solutions at concentrations of 2 x 10^{-5} M (**L**) and 1 x 10^{-5} M (Ag(I) complex).

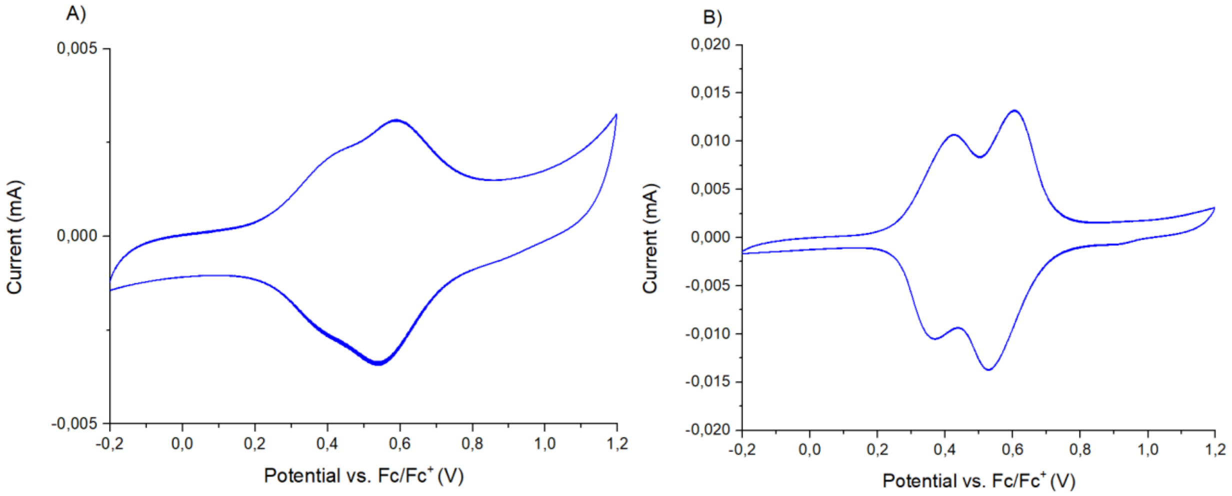

Fig. S3. CVs of **poly-L (A)** and **poly-Ag (B)** obtained at scan rate from 100 mV/s.

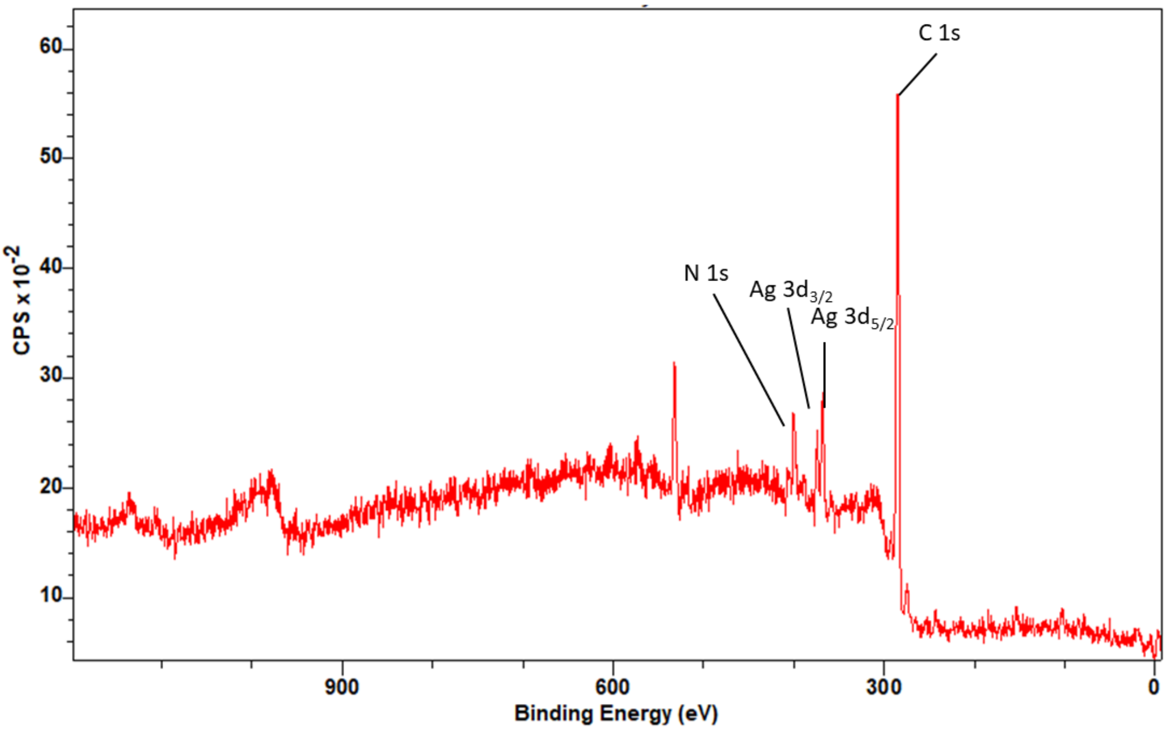

Fig. S4. High-resolution XPS survey spectra of Ag(I) complex.

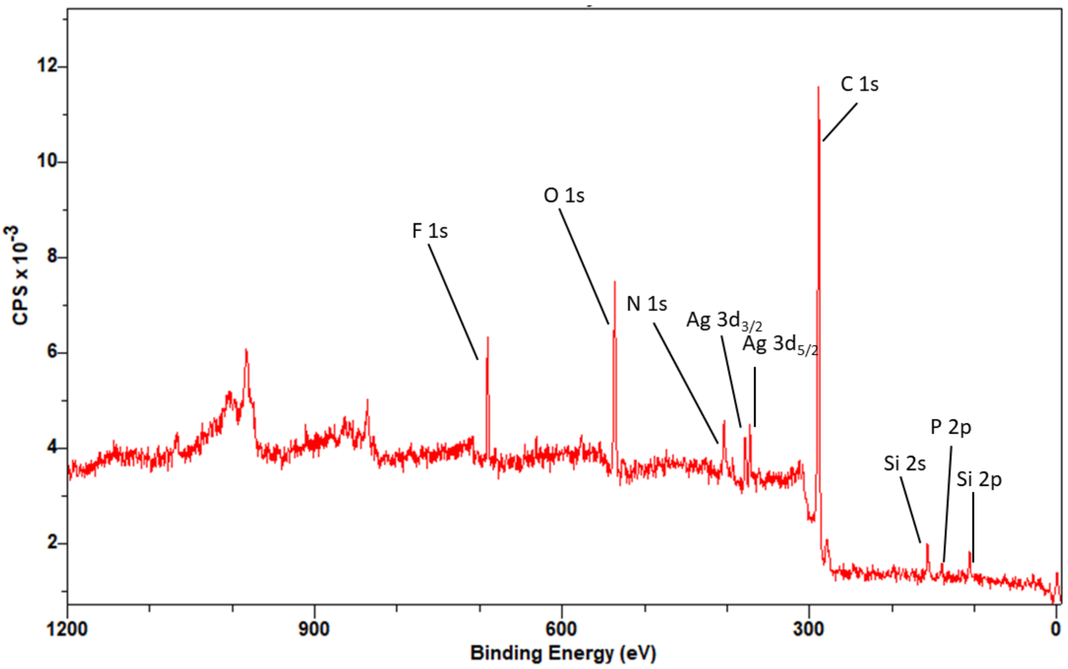

Fig. S5. High-resolution XPS survey spectra of **poly-Ag**.

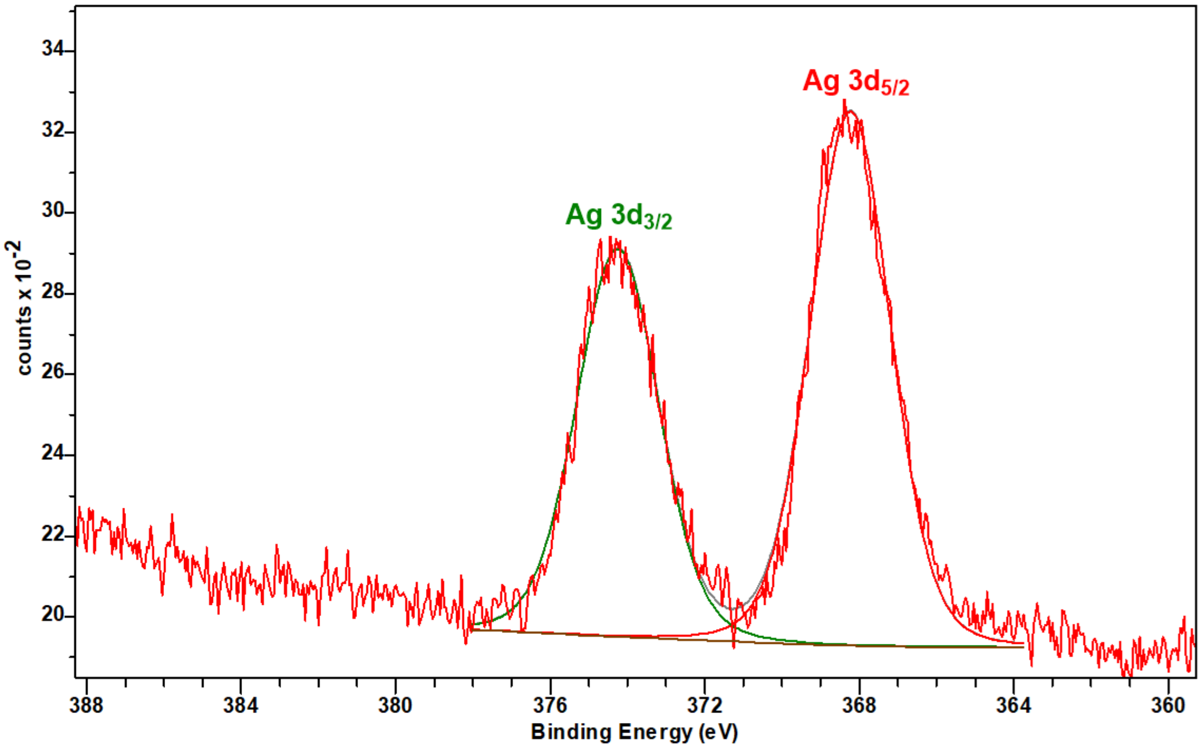

Fig. S6. High-resolution XPS spectra of Ag 3d core levels for Ag(I) complex.

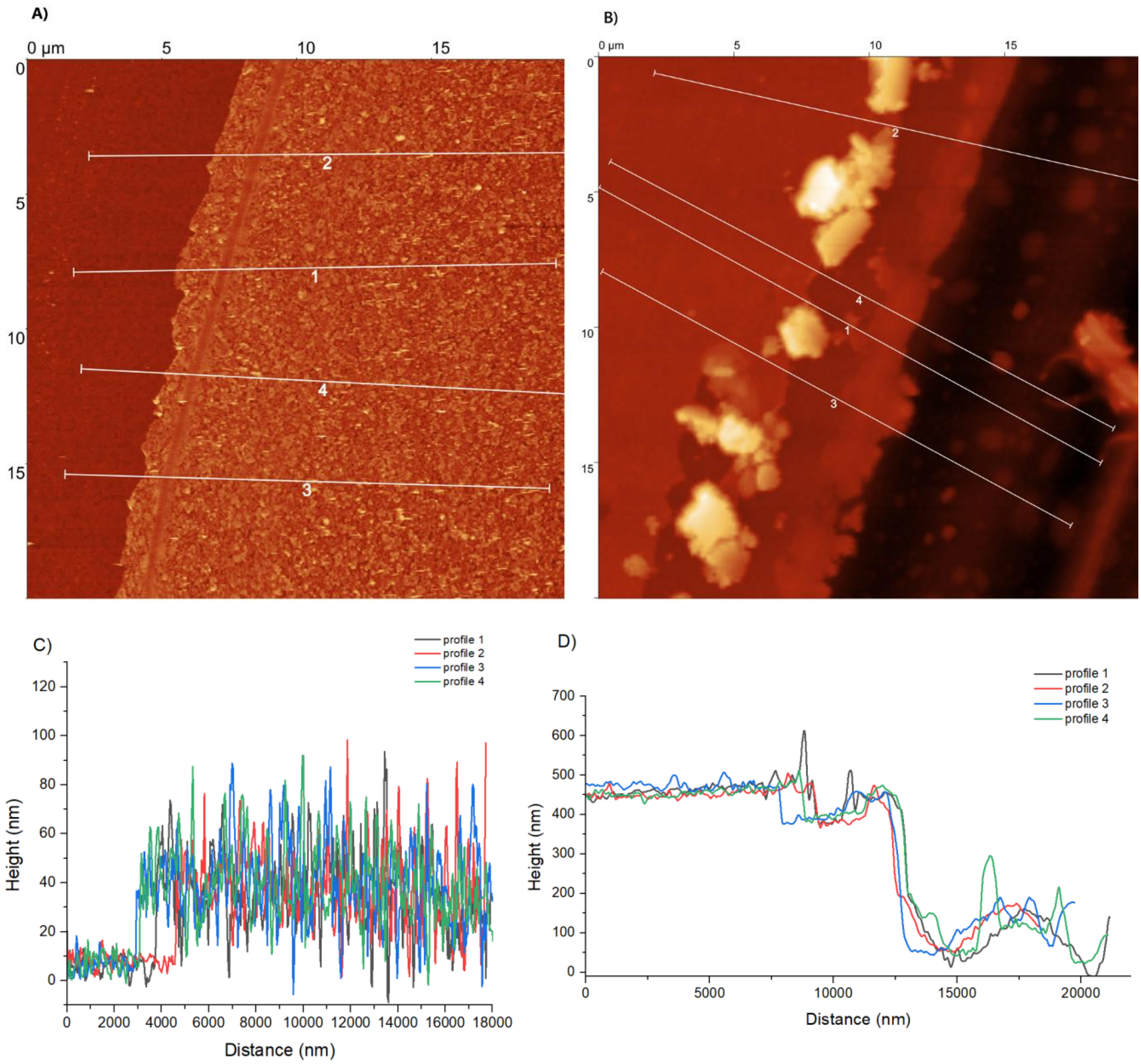

Fig. S7. AFM micrographs of **poly-L** (A) and **poly-Ag** (B) polymer with marked locations for cross-section profile measurements. C,D) plots of layer thickness of **poly-L** and **poly-Ag**.

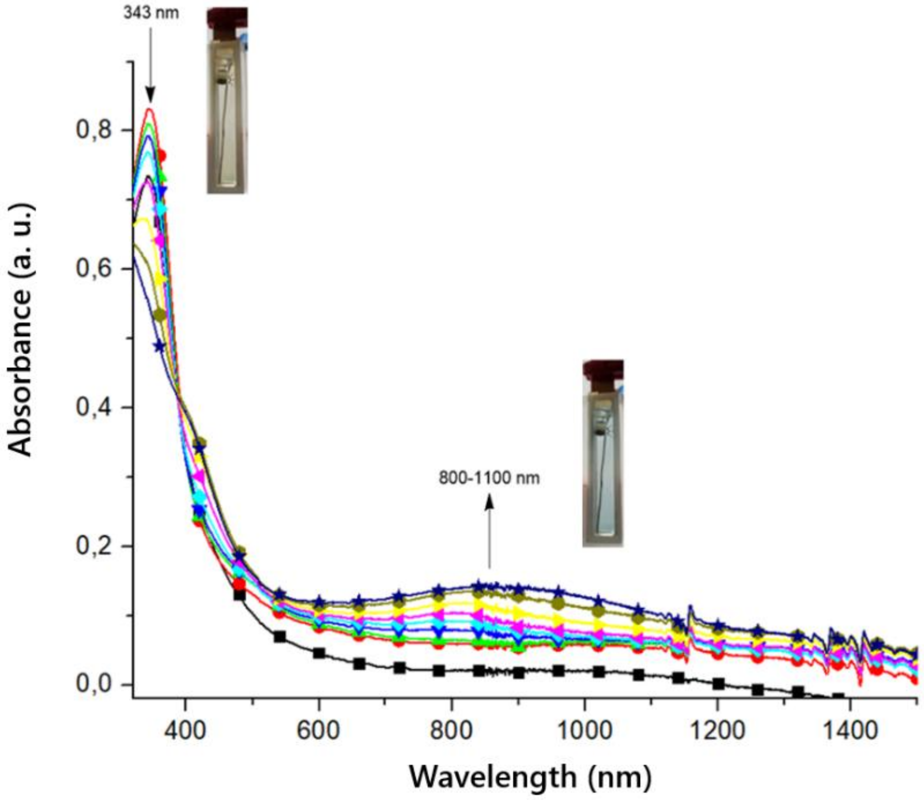

Fig. S8. Spectroelectrochemistry of **poly-L** measured in anhydrous and deaerated 0.1 M solution of $TBAPF_6$ in dichloromethane as a supporting electrolyte versus Fc/Fc^+ with photographs of appropriate polymer oxidized state. Applied potentials: 0.2 V (—■—), 0.4 V (—●—), 0.5 V (—▲—), 0.6 V (—▼—), 0.7 V (—◆—), 0.8 V (—◄—), 0.9 V (—►—), 1.0 V (—●—) and 1.1 V (—★—).

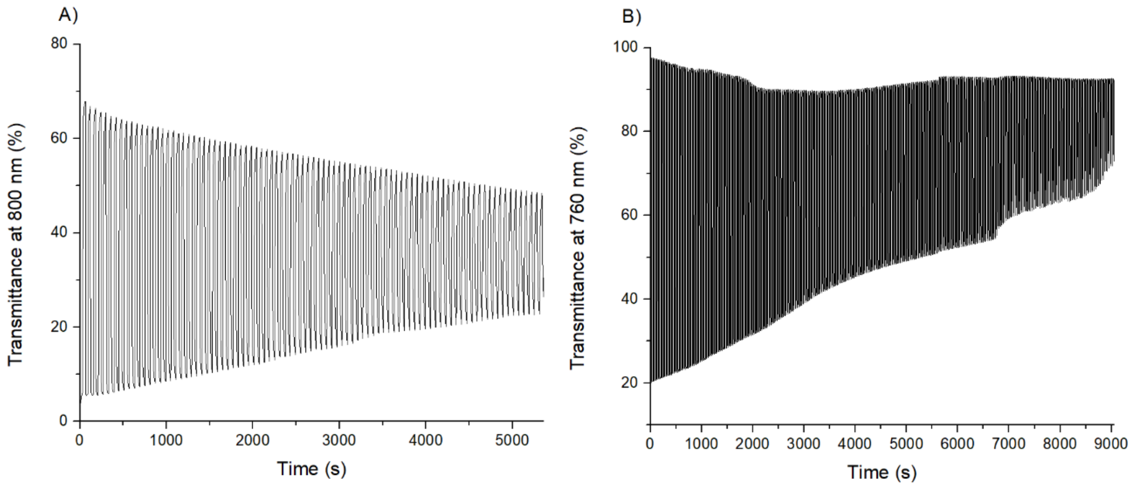

Fig. S9. Electrochromic stability measured between oxidized (0.8 V) and neutral (0 V) form of **poly-L** (A) and **poly-Ag** (B) measured at 800 nm and 760 nm in anhydrous and deaerated 0.1 M dichloromethane:acetonitrile (1:1 *v*/*v*) solution of $LiClO_4$ as a supporting electrolyte by switching between 30 s (A) and 15 s (B) intervals.

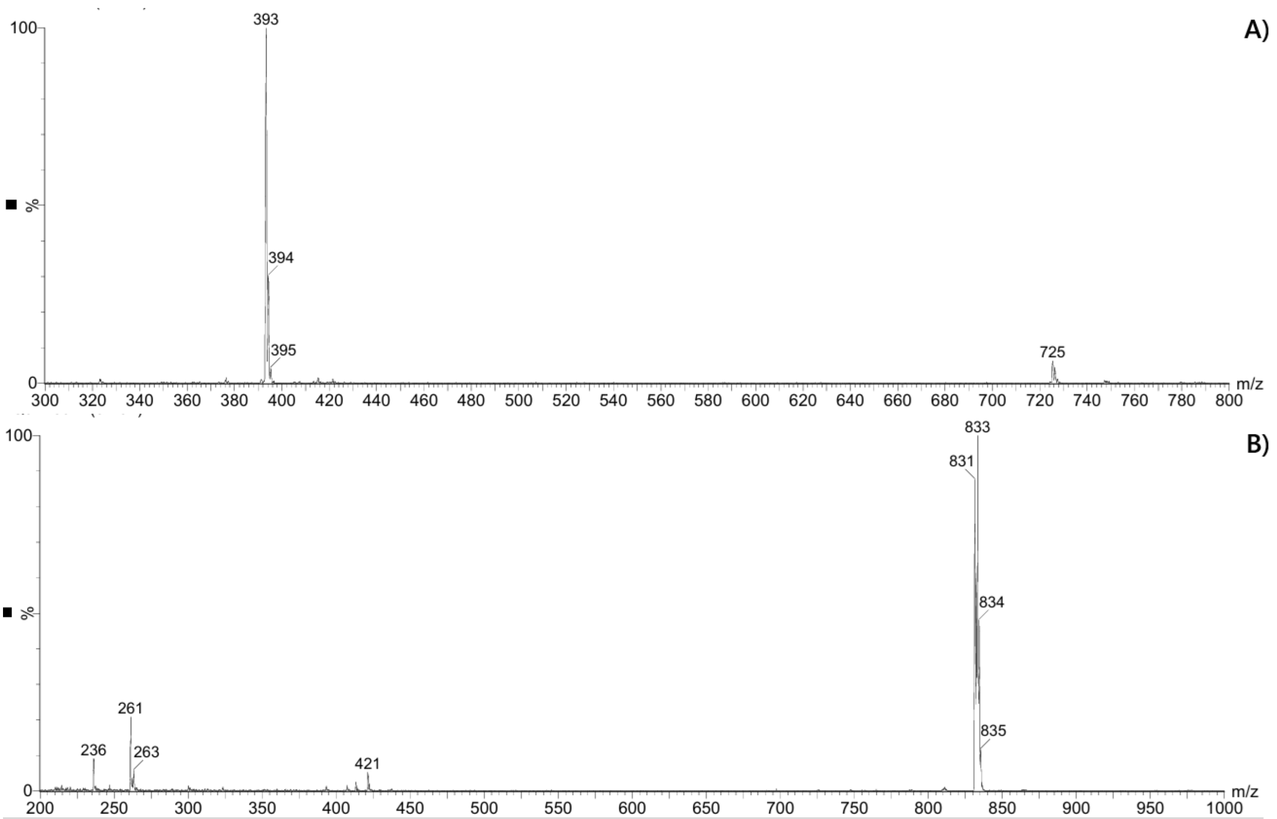

Fig. S10. ESI-MS spectra of **L** (A) and **Ag(I)** complex (B).

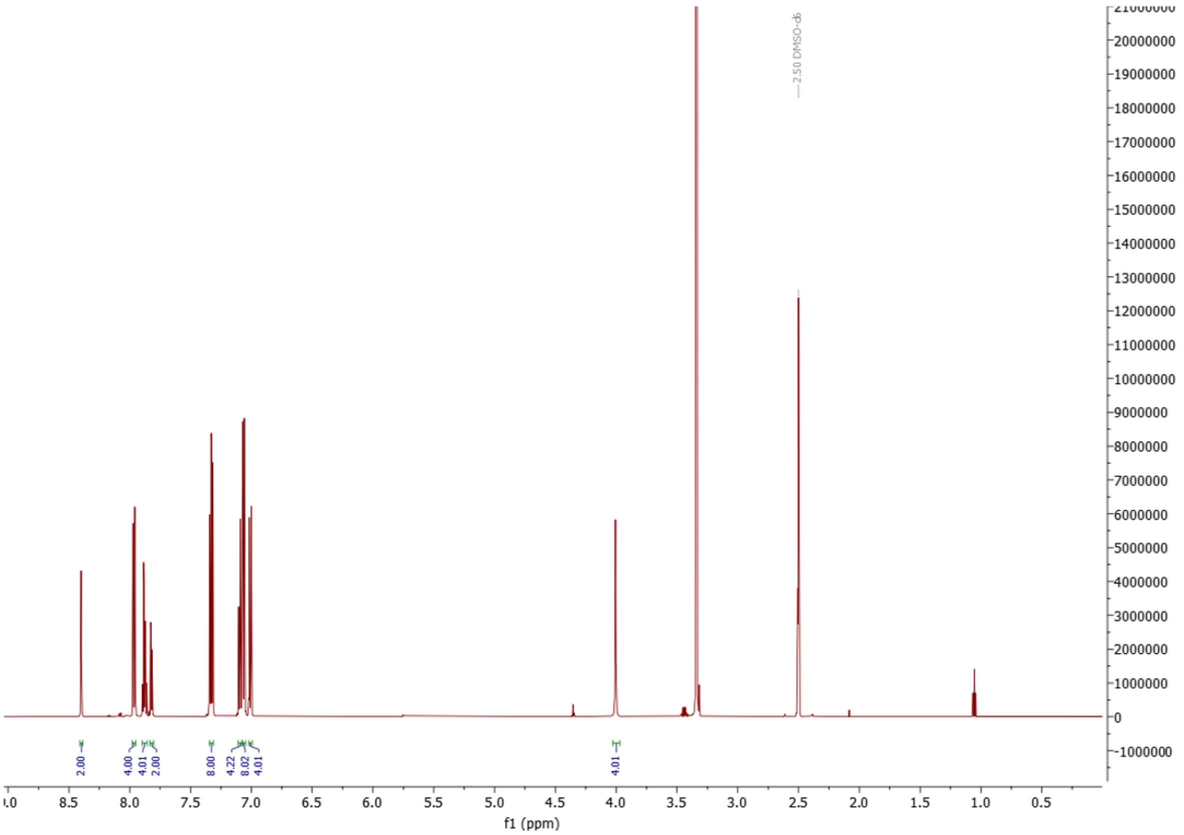

Fig. S11. ^{1}H NMR spectrum of ligand **L**.

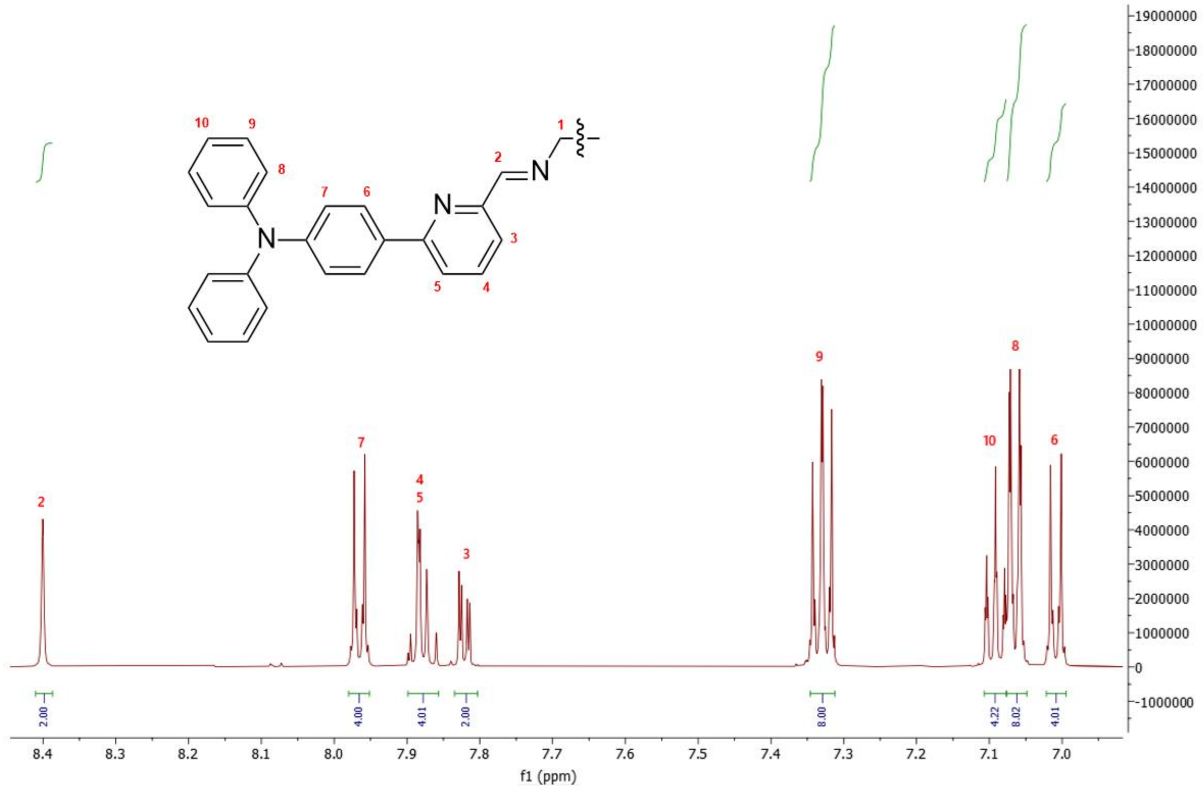

Fig. S12. ^{1}H NMR spectrum of ligand **L** – expanded aromatic region.

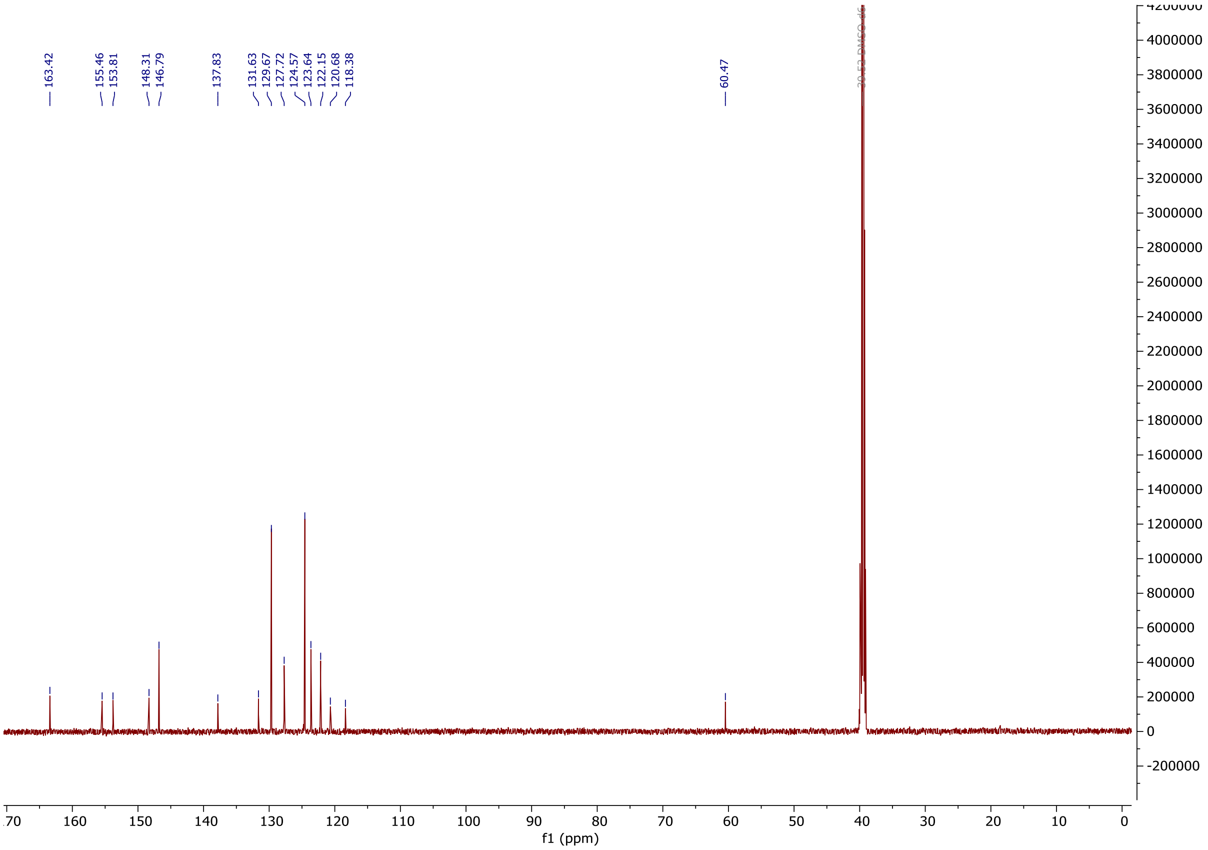

Fig. S13. ^{13}C NMR spectrum of ligand **L**.

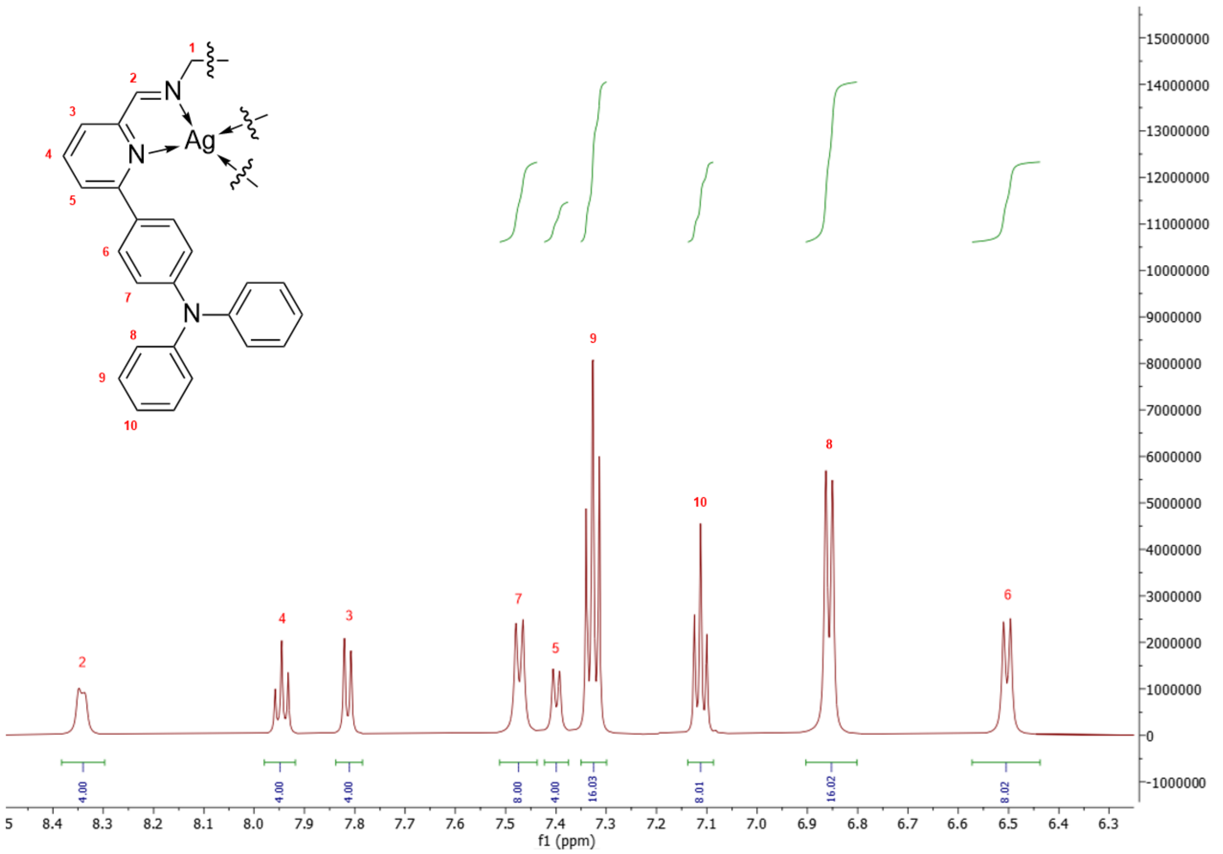

Fig. S14. ^{1}H NMR spectrum of Ag(I) complex – expanded aromatic region.
